# Risk factors for contralateral hip refractures in patients aged over 80 years with intertrochanteric femoral fractures

**DOI:** 10.3389/fsurg.2022.924585

**Published:** 2022-09-13

**Authors:** Shujun Yu, Chen Li, Yuqiao Zhong, Jiacheng Zang, Zhanzhe Zhou, Song Wang, Yinguang Zhang

**Affiliations:** ^1^ The First Department of Hip Traumatology, Tianjin Hospital, Tianjin, China; ^2^ Department of Radiology, Tianjin Hospital, Tianjin, China; ^3^ Department of Orthopedics, Tianjin Xiqing Hospital, Tianjin, China

**Keywords:** contralateral hip refracture, intertrochanteric fracture, intramedullary nails, elderly patients, refractures

## Abstract

**Purpose:**

The purpose of this study was to identify which of the risk factors would contribute to the contralateral fracture in very elderly patients after intramedullary nail fixation.

**Methods:**

Clinical data of 227 intertrochanteric fracture patients aged 80 years or older were retrospectively reviewed. Intramedullary nails (IMNs) were used on all of the patients. Potential risk factors for contralateral hip refractures were determined using univariate and logistic regression analyses.

**Results:**

Contralateral hip refractures occurred in 11 patients (4.84%). Univariate analysis revealed that age, gender, body mass index, fracture classification, hematocrit, D-dimer, and CRP level were not associated with contralateral fractures (*P *> 0.05). However, neurological diseases, cardiovascular disease, and visual impairments were significantly associated with contralateral fractures (*P *< 0.05). Multivariate analysis further revealed that neurological diseases (OR 4.25, *P *= 0.044) and visual impairments (OR 5.42, *P *= 0.015) were independent risk factors associated with contralateral refractures.

**Conclusion:**

To prevent contralateral refractures, more attention should be paid to elderly intertrochanteric fracture patients with underlying neurological disease and visual impairments.

## Introduction

Hip fractures remain a worldwide epidemic and costly injury in the elderly, and the number of patients will increase significantly in the future (1, 2). Some investigational data have shown that between 1.2% and 9% of patients who have a hip fracture will suffer a contralateral refracture within 1 year (3, 4) and up to 20% in the course of their lives (5–7). Second contralateral fractures are related to significantly higher complication rates, socioeconomic cost, and mortality than the first fractures (8–11).

Associations between several risk factors and contralateral fractures have been reported, including gender, osteoporosis, body mass index (BMI), dementia, diabetes, and heart disease (12, 13). In general, patients aged over 80 years are more susceptible to medical comorbidities and possibly at high risk of contralateral fracture (14, 15). Another characteristic of elderly patients is that advanced age is more strongly associated with the risk of intertrochanteric fractures than femoral neck fractures, and intramedullary nails (IMNs) are recommended for fixation (16–18). Some studies, for example, on femoral neck fractures, have revealed that specific fixation methods are associated with a different risk of second hip fracture (19–22). One possible explanation is that surgical fixation may alter an individual’s gait and subsequently increase the fall risk by changing muscle moment and bone structure at the fracture site (23). Similarly, for intertrochanteric fractures, intramedullary nailing has been shown to alter the strength of hip muscles and the walking gait (24, 25). However, no research has been able to determine the IMN relative risk for contralateral refractures, especially in patients of advanced age. We hypothesized that the incidence and risk factors of these patients might differ from those of the general population. The aim is to explore potential contralateral fracture risk factors for intertrochanteric fracture patients who aged over 80 years and were treated with intramedullary nails.

## Materials and methods

Medical records of 227 eligible patients who had been treated for intertrochanteric fracture in our institution from January 2019 to January 2021 were retrospectively reviewed. In the study, intertrochanteric fractures were classified using AO/OTA criteria (26, 27). The inclusion criteria are as follows: (1) ≥80 years old; (2) intertrochanteric fractures; and (3) stabilized with proximal femur nail antirotation (PFNA). Patients with the following conditions were excluded: (1) hip fractures caused by high-energy trauma; (2) open fractures; (3) pathological fractures caused by bone tumors; and (4) incomplete clinical information. The involvers were monitored until a contralateral hip fracture occurred until February 2022. The study was reviewed and approved by the Ethics Committee of our institution.

Various parameters were analyzed to identify potential risk factors for contralateral refractures. The following clinical information is carefully extracted from their clinical data: age, gender, body height/weight, BMI, living circumstances, fracture site, and classification. Comorbidities are categorized as follows: hypertension, cardiovascular disease, diabetes mellitus, respiratory disease, neurological diseases, and visual impairments. The category of cardiovascular disease included coronary atherosclerotic heart disease, cardiomyopathy, heart failure, and arrhythmia. Respiratory diseases included bronchiectasis, pulmonary tuberculosis, chronic obstructive obstructive pulmonary disease (COPD), chronic bronchitis, and bronchial asthma. The category of neurological diseases included dementia, Parkinson’s disease, intracerebral hemorrhage, and stroke. Cataract, diabetic retinopathy, retinal neurodegeneration, and glaucoma are all examples of visual impairments. Surgical information included time from fracture to surgery, operation time, and intraoperative blood loss (ml). Peripheral blood samples were collected for laboratory tests including hematocrit (HCT), hemoglobin levels, D-dimer, and C-reactive protein (CRP).

### Statistical analysis

Continuous data were presented as mean ± standard; categorical data were expressed as frequencies. Statistical analyses were performed using Student’s *t*-test or *χ*^2^ test relatively. Multivariate analysis was performed using logistic regression analysis to determine the risk factors, and results were presented as the odds ratios (OR) by 95% confidence interval (CI). *P *< 0.05 was considered statistically significant.

## Results

The general clinical features of the two groups are presented in Table 1. A total of 234 patients were enrolled in the study; 7 patients were excluded due to a lack of data on whether a contralateral hip fracture occurred. A total of 227 individuals were finally included, including 75 males and 152 females. Contralateral hip refractures occurred in 11 patients (4.84%) within 1 year after the surgery, including 1 male patient and 10 female patients. Each of the 11 patients had a history of falling and sustaining an injury.

**Table 1:** Comparison of baseline data between the two groups.

Characteristics	Non-refractured (*n* = 216)	Refractured (*n* = 11)	*t*/*χ*^2^ value	*P*-value
Age, years (SD)	83.63 ± 3.25	83.27 ± 2.83	0.440	0.725
Gender (male/female)	74/142	1/10		0.106
Body height (cm)	163.3 (7.49)	162.4 (8.64)	0.422	0.674
Body weight (kg)	63.74	66.27	0.668	0.505
Body mass index (kg/m^2^)	23.80 ± 3.76	25.07 ± 3.82	1.085	0.279
Living circumstances				
Assisted living	216 (100%)	11 (100%)		>0.999
Other	0	0		
Fracture site				0.117
Right	101 (46.76%)	2 (18.18%)		
Left	115 (53.24%)	9 (81.82%)		
AO/OTA classification				0.665
A1.1–A1.3	54 (25.00%)	2 (18.18%)		
A2.1–A2.3	140 (64.81%)	7 (63.64%)		
A3.1–A3.3	22 (10.19%)	2 (18.18%)		
Time from fracture to surgery (days)	4.54 ± 3.04	5.27 ± 2.97	0.783	0.434
Operation time (min)	90.69 ± 28.27	87.27 ± 36.63	0.386	0.700
Intraoperative blood loss (ml)	283.02 ± 155.06	277.2 ± 108.08	0.121	0.904

The baseline data from the two groups were compared. No significant differences were found in age, gender, BMI, fracture site, AO/OTA classification, time from fracture to surgery, operation time, and intraoperative blood loss between the contralateral fracture and nonfractured patients (*P *> 0.05).

There was no significant statistical difference between the 11 patients and the 216 controls when preoperative and postoperative laboratory tests of hematocrit, D-dimer level, and C-reactive protein level were examined (*P *> 0.05; Table 2). In addition, no statistical difference was founded in hemoglobin levels between the contralateral fracture and nonfractured patients (*P *> 0.05).

**Table 2:** Comparison of laboratory tests between the two groups.

Characteristics	Nonrefractured (*n* = 216)	Refractured (*n* = 11)	*t* value	*P*-value
Hematocrit				
Preoperation	0.34 ± 0.05	0.33 ± 0.04	0.832	0.406
Postoperation	0.34 ± 0.07	0.31 ± 0.04	1.494	0.137
Hemoglobin levels	118.32 ± 17.48	110.36 ± 14.59	1.483	0.139
D-dimer				
Preoperation	1207.77 ± 1350.00	1227.27 ± 925.30	0.0473	0.962
Postoperation	677.26 ± 567.22	581.82 ± 315.65	0.553	0.581
C-reactive protein				
Preoperation	39.15 ± 36.70	43.36 ± 49.10	0.365	0.716
Postoperation	54.59 ± 38.57	65.82 ± 42.73	0.937	0.350

For comorbid medical diseases, contralateral fracture patients had higher rates of hypertension, cardiovascular disease, neurological diseases, respiratory disease, and visual impairments than the control group (Table 3). However, only visual impairments and neurological and cardiovascular diseases were seen as significantly different between the two groups (*P *< 0.05).

**Table 3:** Comparison of comorbidity between the two groups.

	Nonrefractured (*n* = 216)	Refractured (*n* = 11)	*P*-value
Hypertension	117 (54.17%)	8 (72.73%)	0.353
Cardiovascular disease	49 (22.69%)	6 (54.55%)	0.026
Diabetes mellitus	58 (26.85%)	2 (18.18%)	0.732
Respiratory diseases	26 (12.04%)	3 (27.27%)	0.153
Neurological diseases	36 (16.67%)	5 (45.45%)	0.030
Visual impairments	32 (14.81%)	5 (45.45%)	0.019

Univariate analysis revealed that demographic characteristics, fracture features, and laboratory tests were not associated with contralateral fractures. However, neurological diseases, cardiovascular disease, and visual impairments were significantly associated with contralateral fractures. Multivariate analysis further revealed that visual impairments (OR 5.42, *P *= 0.015) and neurological diseases (OR 4.25, *P *= 0.044) were independent risk factors for contralateral hip refractures (Table 4).

**Table 4:** Univariate and multivariate analyses of factors.

	OR	95% CI	*P*-value
Cardiovascular disease	2.53	0.63–9.98	0.177
Neurological diseases	4.25	1.02–18.17	0.044
Visual impairments	5.42	1.35–22.16	0.015

## Discussion

Contralateral hip refractures are associated with major clinical and social cost implications (4, 11, 28, 29). How to develop effective preventive strategies for hip fracture patients is still under controversy (13). Recently, reports have raised the question of whether specific surgical fixation of the initial hip fracture is associated with a different risk of subsequent contralateral fracture. Souder et al. (22) found an increased risk of hip refractures in patients who underwent closed reduction and percutaneous puncture compared to those who underwent arthroplasty. Changes in individual’s gait and subsequent fall risk due to different fixation methods may be one of the important reasons (23, 24). To our knowledge, no research has determined the IMN relative risk of contralateral hip refractures, especially in patients of advanced age.

Our results showed that 11 in 227 patients (4.84%) suffered a contralateral hip refracture during the data collection period. The incidence correlated with the risk of 3%–10% for second hip refractures (3, 20, 30, 31). In general, elderly patients are prone to contralateral fractures due to osteoporosis and susceptible to medical comorbidities (15). However, only a few studies have reported the age-specific incidence and risk factors for contralateral fractures. Yamanashi et al. (32) reported that the incidence was 3.8% within the first year in patients aged ≥65 years. Similarly, Lönnroos et al. (33) noted an incidence of 5.08% for patients aged ≥60 years within the first year, and the rate increased further to 8.11% at 2 years following the initial fracture. For very elderly patients, Vochteloo et al. (34) found that the incidence of patients aged over 85 years was not different from other age categories. Lawrence et al. (35) also found female patients over the age of 84 years have a similar risk to the general population. Similarly, our results showed that the incidence of patients aged over 80 years was not significantly increased compared to previous hip refracture data. One theoretical explanation is that the increasing age of patients is not exclusively related to bone strength loss. Indeed, Gnudi et al. (36) suggested that bone loss will gradually slow down after the age of 65 years. Moreover, we noted that none of the enrolled patients lived alone and had a reduced range of physical activity, which may have reduced the risk of falls, which are a major cause of hip fractures.

To further identify risk factors, we evaluated the difference between the patients who suffered a contralateral hip fracture and those in the control group. Univariate analysis revealed that gender distribution, BMI, fracture classification, operation time, and intraoperative blood loss were not associated with contralateral fractures. Although some authors have emphasized the relationship between gender and second hip fractures (7, 31), our study did not support this suggestion. Similar to our results, no significant gender difference was also seen in previous studies (15, 33, 34, 37, 38).

At present, few publications have emphasized the value of laboratory-based indicators for contralateral fracture risk. Preoperative CRP was found to be a primary risk factor for postoperative death in elderly patients with hip fractures in a recent study (39). Chen et al. (37) reported that the serum CRP/Alb ratio is a risk factor in elderly hip fracture patients treated by total hip arthroplasty. In our study, there was no difference between the patient’s preoperative and postoperative laboratory tests of hematocrit, D-dimer level, and CRP level between the two groups. Traumatic stress and perioperative drugs all affect the level of expression of these inflammatory and nutritional indicators in the perioperative period (40); therefore, their significance in contralateral fracture needs to be further evaluated.

An increased risk of contralateral hip refractures has been found to be associated with several comorbid diseases (12, 13, 38, 41–43), including hypertension, diabetes mellitus, cardiovascular disease, neurological diseases, respiratory diseases, and visual impairments. In our study, neurological diseases and visual impairments were found to be significantly associated with contralateral hip refractures. Although contralateral fracture patients had higher rates of hypertension, cardiovascular disease, and respiratory diseases than the control group, differences were only seen in neurological diseases, cardiovascular disease, and visual impairments using univariate analysis. Multivariate analysis revealed that neurological diseases and visual impairments were independent risk factors for contralateral hip refractures. Risk factors determined in our study can aid in identifying high-risk populations among very elderly intertrochanteric fracture patients. However, our research also has some limitations. Some clinical information was collected retrospectively, and a relatively small population was the study's main limitation. This may have led to a bias in the analysis of the incidence of contralateral fractures. In addition, some potentially meaningful items, such as the clinical data on vitamin D levels and the use of bone health medications (vitamin D, bisphosphonates, trospium) were not available for all patients, so this was not analyzed in the study.

In summary, neurological diseases and underlying visual impairments are risk factors for contralateral hip refractures in intertrochanteric fracture patients aged over 80 years and who were treated with intramedullary nails. More attention should be given to the patients with these underlying comorbidities.

## Data Availability

The original contributions presented in the study are included in the article/Supplementary Material, further inquiries can be directed to the corresponding author/s.

## References

[1] Rapp K Büchele G Dreinhöfer K Bücking B Becker C Benzinger P. Epidemiology of hip fractures. Z Gerontol Geriat. (2019) 52:10–6. 10.1007/s00391-018-1382-z PMC6353815 29594444

[2] van Leendert JAA Linkens AEMJH Poeze M Pijpers E Magdelijns F Ten Broeke RHM Mortality in hip fracture patients after implementation of a nurse practitioner-led orthogeriatric care program: results of a 1-year follow-up. Age Ageing. (2021) 50:1744–50. 10.1093/ageing/afab031 33710294

[3] Chang J-D Yoo J-H Reddy P Lee S-S Hwang J-H Kim T-Y. Risk factors for contra-lateral hip fracture in elderly patients with previous hip fracture. Injury. (2013) 44:1930–3. 10.1016/j.injury.2013.03.034 23688407

[4] Ho AWH Wong SH. Second hip fracture in Hong Kong - incidence, demographics, and mortality. Osteoporos Sarcopenia. (2020) 6:71–4. 10.1016/j.afos.2020.05.004 32715097 PMC7374255

[5] Berry SD Samelson EJ Hannan MT McLean RR Lu M Cupples LA Second hip fracture in older men and women: the framingham study. Arch Intern Med. (2007) 167:1971–6. 10.1001/archinte.167.18.1971 17923597

[6] Chapurlat RD Bauer DC Nevitt M Stone K Cummings SR. Incidence and risk factors for a second hip fracture in elderly women. The study of osteoporotic fractures. Osteoporos Int. (2003) 14:130–6. 10.1007/s00198-002-1327-6 12730779

[7] Lau JCF Ho KW Sadiq S. Patient characteristics and risk of subsequent contralateral hip fracture after surgical management of first fracture. Injury. (2014) 45:1620–3. 10.1016/j.injury.2014.05.030 24947502

[8] Müller F Galler M Zellner M Bäuml C Roll C Füchtmeier B. Comparative analysis of non-simultaneous bilateral fractures of the proximal femur. Eur J Trauma Emerg Surg. (2019) 45:1053–7. 10.1007/s00068-018-0981-0 30014273

[9] Sawalha S Parker MJ. Characteristics and outcome in patients sustaining a second contralateral fracture of the hip. J Bone Joint Surg Br. (2012) 94:102–6. 10.1302/0301-620X.94B1.27983 22219256

[10] Sobolev B Sheehan KJ Kuramoto L Guy P. Excess mortality associated with second hip fracture. Osteoporos Int. (2015) 26:1903–10. 10.1007/s00198-015-3104-3 25910745

[11] van der Steenhoven TJ Staffhorst B Van de Velde SK Nelissen RGHH Verhofstad MHJ. Complications and institutionalization are almost doubled after second hip fracture surgery in the elderly patient. J Orthop Trauma. (2015) 29:e103–8. 10.1097/BOT.0000000000000233 25210832

[12] Zhu Y Chen W Sun T Zhang Q Cheng J Zhang Y. Meta-analysis of risk factors for the second hip fracture (SHF) in elderly patients. Arch Gerontol Geriatr. (2014) 59:1–6. 10.1016/j.archger.2014.02.012 24657007

[13] Liu S Zhu Y Chen W Sun T Cheng J Zhang Y. Risk factors for the second contralateral hip fracture in elderly patients: a systematic review and meta-analysis. Clin Rehabil. (2015) 29:285–94. 10.1177/0269215514542358 25027445

[14] Maruhashi T Kajikawa M Kishimoto S Hashimoto H Takaeko Y Yamaji T Vascular function is further impaired in subjects aged 80 years or older. Hypertens Res. (2020) 43:914–21. 10.1038/s41440-020-0435-z 32269307

[15] Angthong C Suntharapa T Harnroongroj T. Major risk factors for the second contralateral hip fracture in the elderly. Acta Orthop Traumatol Turc. (2009) 43:193–8. 10.3944/AOTT.2009.193 19717935

[16] Karagas MR Lu-Yao GL Barrett JA Beach ML Baron JA. Heterogeneity of hip fracture: age, race, sex, and geographic patterns of femoral neck and trochanteric fractures among the US elderly. Am J Epidemiol. (1996) 143:677–82. 10.1093/oxfordjournals.aje.a008800 8651229

[17] Baudoin C Fardellone P Sebert JL. Effect of sex and age on the ratio of cervical to trochanteric hip fracture. A meta-analysis of 16 reports on 36,451 cases. Acta Orthop Scand. (1993) 64:647–53. 10.3109/17453679308994590 8291411

[18] Niu E Yang A Harris AHS Bishop J. Which fixation device is preferred for surgical treatment of intertrochanteric hip fractures in the United States? A survey of orthopaedic surgeons. Clin Orthop Relat Res. (2015) 473:3647–55. 10.1007/s11999-015-4469-5 26208608 PMC4586189

[19] Shabat S Gepstein R Mann G Kish B Fredman B Nyska M. The second hip fracture–an analysis of 84 elderly patients. J Orthop Trauma. (2003) 17:613–7. 10.1097/00005131-200310000-00003 14574188

[20] Dretakis KE Dretakis EK Papakitsou EF Psarakis S Steriopoulos K. Possible predisposing factors for the second hip fracture. Calcif Tissue Int. (1998) 62:366–9. 10.1007/s002239900446 9504964

[21] Yoshii I Kitaoka K Hashimoto K. Clinical characteristics of osteoporotic second hip fracture: from the data of clinical pathway with regional alliance in rural region in Japan. J Orthop Sci. (2019) 24:836–41. 10.1016/j.jos.2018.12.029 30772124

[22] Souder CD Brennan ML Brennan KL Song J Williams J Chaput C. The rate of contralateral proximal femoral fracture following closed reduction and percutaneous pinning compared with arthroplasty for the treatment of femoral neck fractures. J Bone Joint Surg Am. (2012) 94:418–25. 10.2106/JBJS.J.01134 22398735

[23] Zlowodzki M Ayeni O Ayieni O Petrisor BA Bhandari M. Femoral neck shortening after fracture fixation with multiple cancellous screws: incidence and effect on function. J Trauma. (2008) 64:163–9. 10.1097/01.ta.0000241143.71274.63 18188116

[24] Güven M Kocadal O Akman B Poyanlı OS Kemah B Atay EF. Proximal femoral nail shows better concordance of gait analysis between operated and uninjured limbs compared to hemiarthroplasty in intertrochanteric femoral fractures. Injury. (2016) 47:1325–31. 10.1016/j.injury.2016.03.009 27017452

[25] Noda M Saegusa Y Takahashi M Takada Y Fujita M Shinohara I. Decreased postoperative gluteus medius muscle cross-sectional area measured by computed tomography scan in patients with intertrochanteric fractures nailing. J Orthop Surg. (2017) 25:2309499017727943. 10.1177/2309499017727943 28920547

[26] Orthopaedic Trauma Association Committee for Coding and Classification. Fracture and dislocation compendium. J Orthop Trauma. (1996) v–ix(10 Suppl 1):1–154. PMID: 8814583

[27] Marsh JL Slongo TF Agel J Scott Broderick J Creevey W DeCoste TA Fracture and dislocation classification compendium 2007: Orthopaedic Trauma Association classification, database and outcomes committee. J Orthop Trauma. (2007) 21:S1–S6. 10.1097/00005131-200711101-00001 18277234

[28] Hagino H. Current and future burden of hip and vertebral fractures in Asia. Yonago Acta Med. (2021) 64:147–54. 10.33160/yam.2021.05.001 34025188 PMC8128659

[29] Mazzucchelli R Pérez-Fernández E Crespí N García-Vadillo A Rodriguez Caravaca G Gil de Miguel A Second hip fracture: incidence, trends, and predictors. Calcif Tissue Int. (2018) 102:619–26. 10.1007/s00223-017-0364-2 29159516

[30] Kaper BP Mayor MB. Incidence of bilateral proximal femoral fractures in a tertiary care center. Orthopedics. (2001) 24:571–4. 10.3928/0147-7447-20010601-14 11430737

[31] Gaumetou E Zilber S Hernigou P. Non-simultaneous bilateral hip fracture: epidemiologic study of 241 hip fractures. Orthop Traumatol Surg Res. (2011) 97:22–7. 10.1016/j.otsr.2010.07.011 21239241

[32] Yamanashi A Yamazaki K Kanamori M Mochizuki K Okamoto S Koide Y Assessment of risk factors for second hip fractures in Japanese elderly. Osteoporos Int. (2005) 16:1239–46. 10.1007/s00198-005-1835-2 15729479

[33] Lönnroos E Kautiainen H Karppi P Hartikainen S Kiviranta I Sulkava R. Incidence of second hip fractures. A population-based study. Osteoporos Int. (2007) 18:1279–85. 10.1007/s00198-007-0375-3 17440675

[34] Vochteloo AJH Borger van der Burg BLS Röling MA van Leeuwen DH van den Berg P Niggebrugge AHP Contralateral hip fractures and other osteoporosis-related fractures in hip fracture patients: incidence and risk factors. An observational cohort study of 1,229 patients. Arch Orthop Trauma Surg. (2012) 132:1191–7. 10.1007/s00402-012-1520-9 22526197 PMC3400761

[35] Lawrence TM Wenn R Boulton CT Moran CG. Age-specific incidence of first and second fractures of the hip. J Bone Joint Surg Br. (2010) 92:258–61. 10.1302/0301-620X.92B2.23108 20130319

[36] Gnudi S Sitta E Fiumi N. Bone density and geometry in assessing hip fracture risk in post-menopausal women. Br J Radiol. (2007) 80:893–7. 10.1259/bjr/37401526 17875597

[37] Chen L Zhang J Zhang W Deng C. Correlation between C-reactive protein/albumin and contralateral hip refracture after total hip arthroplasty in elderly patients with hip fractures. Ann Palliat Med. (2020) 9:1055–61. 10.21037/apm-20-855 32434368

[38] Juhász K Boncz I Patczai B Mintál T Sebestyén A. Risk factors for contralateral hip fractures following femoral neck fractures in elderly: analysis of the Hungarian nationwide health insurance database. Eklem Hastalik Cerrahisi. (2016) 27:146–52. 10.5606/ehc.2016.30 27902169

[39] Kim B-G Lee Y-K Park H-P Sohn H-M Oh A-Y Jeon Y-T C-reactive protein is an independent predictor for 1-year mortality in elderly patients undergoing hip fracture surgery: a retrospective analysis. Medicine. (2016) 95:e5152. 10.1097/MD.0000000000005152 27787371 PMC5089100

[40] Eriksson AL Movérare-Skrtic S Ljunggren Ö Karlsson M Mellström D Ohlsson C. High-sensitivity CRP is an independent risk factor for all fractures and vertebral fractures in elderly men: the MrOS Sweden study. J Bone Miner Res. (2014) 29:418–23. 10.1002/jbmr.2037 23857741 PMC4238816

[41] Fukushima T Sudo A Uchida A. Bilateral hip fractures. J Orthop Sci. (2006) 11:435–8. 10.1007/s00776-006-1056-3 17013728

[42] Lee Y-K Ha Y-C Yoon B-H Koo K-H. Incidence of second hip fracture and compliant use of bisphosphonate. Osteoporos Int. (2013) 24:2099–104. 10.1007/s00198-012-2250-0 23247329

[43] Mitani S Shimizu M Abo M Hagino H Kurozawa Y. Risk factors for second hip fractures among elderly patients. J Orthop Sci. (2010) 15:192–7. 10.1007/s00776-009-1440-x 20358331

